# Comprehensive Analysis of the Relationships Between Tumor Mutation Burden With Immune Infiltrates in Cervical Cell Carcinoma

**DOI:** 10.3389/fmolb.2020.582911

**Published:** 2020-10-06

**Authors:** Cankun Zhou, Chaomei Li, Shunqing Peng, Liangcheng Zhou, Huan Li

**Affiliations:** ^1^Department of Gynecology, Southern Medical University Affiliated Maternal & Child Health Hospital of Foshan, Foshan, China; ^2^School of Medicine, Southern Medical University, Guangzhou, China; ^3^Department of Medical Oncology, Meizhou People’s Hospital (Huangtang Hospital), Meizhou Academy of Medical Sciences, Meizhou Hospital Affiliated to Sun Yat-sen University, Meizhou, China; ^4^Department of Nephrology, Maoming City People’s Hospital Affiliated to Nanfang Medical University, Maoming, China; ^5^Department of gynecology, Peking University Shenzhen Hospital, Shenzhen, China

**Keywords:** cervical cell carcinoma, TCGA, tumor mutation burden, immune cell, immune infiltration

## Abstract

We aimed to investigate the prognosis of tumor mutation burden (TMB) in cervical cell carcinoma (CCC) and its potential association with tumor-infiltrating immune cells. The data from TCGA were analyzed, and higher TMB levels conferred high overall survival time, associated with higher T staging (*p* = 0.006) and older age (*p* = 2.961e−04). Through “CIBERSORT” package and Wilcoxon rank-sum test, the high TMB group exhibited higher levels of infiltration of T cell CD8 (*p* = 0.008), T cell CD4 memory activation (*p* = 0.006), T cell follicular assistance (*p* = 0.018), and Macrophage M1 (*p* = 0.037). In addition, 478 TMB-associated differentially expressed genes were identified, and two hub TMB-associated immune genes were identified, including CLEC3B and COL4A2. The TMB prognostic model (TMBPM) based on two hub immune genes showed robust prognostic capability in both training set and testing sets, and the higher the TMBPM score, the worse the prognosis. Finally, survival time was higher for high CLEC3B expression levels (*p* = 0.038) and lower for high COL4A2 expression levels (*p* = 0.033). Notably, there is an association between the expression of these two genes and immune infiltration in CCC. CLEC3B expression was most significantly positively correlated with B cells, CD4+ T cells, and Macrophage infiltration. COL4A2 expression was most significantly positively correlated with the presence of Macrophage and Dendritic cell infiltration. In addition, we observed that CLEC3B and COL4A carry mutations in multiple forms that normally suppress immune infiltration, including B cells, CD8+ T cells, and Macrophages.

## Introduction

About 100 million people worldwide suffer from gynecological cancer, accounting for about 18% of all female cancers (del Carmen et al., 2015). Cervical cell carcinoma (CCC) is still one of the most common malignant tumors in women, ranking fourth after breast, colorectal and lung cancer, and more common in low-and middle-income countries. According to statistics, there are more than 500,000 new cases of CCC worldwide every year, and about 300,000 cases die of CCC every year (Bray et al., 2018). Most of the early CCC can be cured by surgery, the main treatment of locally advanced CCC is simultaneous radiotherapy and chemotherapy, but the treatment of advanced and recurrent CCC is limited, the 5-year survival rate is less than 20%. In addition to traditional surgery, radiotherapy and chemotherapy, anti-angiogenic targeted therapy and tumor immunotherapy have achieved remarkable results. The targeted drug bevacizumab combined with chemotherapy has significantly improved the overall survival of advanced CCC and has been approved for the treatment of advanced CCC (Tewari et al., 2017).

Recently, targeted therapy for various molecular targets in CCC and its tumor microenvironment (mainly immune cells and stromal cells) have been increasingly developed and applied. In these targeted treatments, there is growing evidence that immune checkpoint inhibitors can be used as a feasible potential treatment strategy for CCC (Koh et al., 2019). Antibody immunotherapy represented by immune checkpoint inhibitors has become a research focus in recent years.

At present, there are many clinical trials of immunosuppressive agents in the treatment of CCC, but most of them have not been started for long, and all of them are in phase I or phase II of the trial, and there are not many clinical trials that publish results (Duska et al., 2017; Frenel et al., 2017; Hollebecque et al., 2017; Mayadev et al., 2017; Lheureux et al., 2018). The predictive markers of immunotherapy are the focus of clinicians and patients, but in the clinical trials of immunotherapy for CCC, there are no other markers related to the efficacy of immunotherapy except Cytotoxic T lymphocyte-associated molecule-4 (CTLA-4) and programmed cell death receptor-1 (PD-1) (Dyer et al., 2019). Tumor mutation burden (TMB) is becoming a new biomarker, especially predicting the response to PD-L1 therapy. However, only about one-fifth of cancer patients currently benefit from immunotherapy (Braun et al., 2016), so it is important to explore the molecular mechanisms of immunotherapy responsiveness to tumor immunotherapy.

Wang showed that the prognostic effect of TMB and the relationship between TMB and immune infiltration varies with different types of cancer (Wang and Li, 2019). There are few studies on TMB with immune infiltrates in CCC, so we tried to investigate the prognostic role of TMB and the potential association between TMB and immune infiltration through the Cancer Genome Atlas (TCGA). Our findings may provide more information about TMB and immune infiltration with CCC.

## Materials and Methods

### Materials and Data Pre-processing

Cervical cell carcinoma data was download from the TCGA^1^ on May 01, 2019. Download data includes mutation files of the Simple Nucleotide Variation, clinical date (futime, fustat, age, grade, TMN) and RNA-seq gene expression profiles. Mutation information was available on 289 samples, clinical information was available on 307 TCGA CCC samples, and RNA-seq data was available on 309 samples.

The somatic mutation data in varscan.somatic.maf format was extracted from the downloaded Masked Somatic Mutation file, and then we use R 3.5.3^2^, Bioconductor v3.9^3^ (Huber et al., 2015), and MAFtools v1.8.10 (Mayakonda and Koeffler, 2016) visualize the mutations. For mutation burden estimation, the total mutation frequency of each sample was calculated first, the human exon length was set at 38 Mb, and the estimated mutant tumor burden (TMB) of each sample was (total mutation frequency/38).

### Integrated Analysis of the Relationship Between TMB and Survival and Clinical Characters

We performed survival analyses of CCC patients based on TMB values, and the associations of high TMB/low TMB and corresponding disease-free survival were analyzed by Kaplan-Meier method (R package survival v2.44-1.1) and evaluated using log-rank test. Regarding the relationship between TMB and clinical characters, we used the Wilcox rank-sum test to calculate the significance of TMB differences between younger and elderly patients with a threshold of *p*-value b 0.05, and the Kruskal–Wallis test to calculate the significance of TMB differences between patients grouped by Tumor (Topography) with a threshold of *p*-value < 0.05.

### Functional Analysis of TMB-Related DE Genes

To understand the relationship between TMB and gene expression, we first grouped RNA-seq gene expression samples according to the TMB median value, which were divided into high-TMB subtype and low-TMB subtype, and we performed DEG analysis using R package “limma v3.38.3” (Ritchie et al., 2015). We standardized TCGA CCC gene expression data based on −2 logarithmic transformation, and used Wilcox rank-sum test for DE gene analysis. *p*-value (*p* < 0.05) was considered statistically significant for DE mRNA. Finally, all the differentially expressed genes were output by *p* < 0.05 and |logFC| > 0.5. Heatmaps and clustering were generated using R package “pheatmap v1.0.12.”

Functional enrichment analysis and visualization of DE Genes was performed by R package “clusterProfiler v3.10.1, org.Hs.eg.db v3.7.0, enrichplot v1.2.0, ggplot2 v3.1.1” (Xu et al., 2018; Yu, 2018) to identify Gene Ontology (GO) categories by their biological processes (BP), molecular functions (MF), or cellular components (CC). The R packages were also used to perform Kyoto Encyclopedia of Genes and Genomes (KEGG) pathways enrichment analysis and visualization of DE Genes. *p*-value < 0.05 and *q*-value < 0.05 were used as the cut-off. Finally, through the String online database and Cytoscape software, analyzed and constructed a protein–protein interaction (PPI) network to search for functional correlation between proteins.

### Relationship Between the Tumor Mutation Burden and Immune Infiltration

The CIBERSORT tool uses the deconvolution of large amounts of gene expression data and a complex algorithm for calculating many immune cell types in heterogeneous samples as tumor matrix. High resolution is a key advantage of CIBERSORT, which also lists 22 immune cell types and applies the characteristics of ∼500 marker genes to quantify the relative fraction of each cell type (Newman et al., 2015). The method was successfully verified by FACS and used to determine the immune cell landscape in several in several malignancies, such as the breast, liver, colorectal (Ali et al., 2016; Rohr-Udilova et al., 2018; Xiong et al., 2018). Here, the transcriptome data of TCGA CCC were corrected with package “limma,” and then the immune cell content matrix was obtained by CIBERSORT’s deconvolution algorithm, with *p*-value < 0.05 as the cut-off. Then, the immune cell content matrix samples were divided into low-TMB subtype and high-TMB subtype according to the median value of TMB. Finally, R package “vioplot v0.3.0” was used to visually draw the violin diagram for each immune cell, and we used the Wilcox rank-sum test to calculate whether the content of each tumor cell in simples between high- and low-TMB subtypes was statistically significant.

### Development and Validation of the Immune Prognostic Signature for CCC

We obtained a list of immune-related genes from the Immport database^4^ and used the “VennDiagram” package to select immune genes that were differentially expressed between the two groups. All CCC patients from TCGA (*n* = 287) used as the training set. We further performed a Cox proportional risk regression analysis of immune infiltrating cells to obtain the respective coefficients (β) of the two hub immune genes, TMB Prognostic model (TMBPM) = EXP_immune–gene1_ × β1 + EXP_immune–gene2_ × β2. Finally, we plotted Receiver Operating Characteristic (ROC) curves using the “survivalROC” package to assess the predictive value of the two immune genes in cervical cancer. To determine the feasibility and reliability of the TMBPM, all cervical cancer patients from TCGA (*n* = 287) were randomly assigned to the testing set I (*n* = 143) and testing set II (*n* = 144), with the same statistical methods as above.

### Immune Infiltrate Analysis

To gain a deeper understanding of the immune aspect, we first used Kaplan-Meier analysis to understand the survival analysis of the hub TMB-related immune genes. Through the TIMER official website^5^, we further evaluated the mutation types of the genes and correlation with tumor infiltration, and to explore correlations between genes. A *p*-value < 0.05 was considered significant.

## Results

### The TCGA CCC Mutation Cohort

**FIGURE 1:**
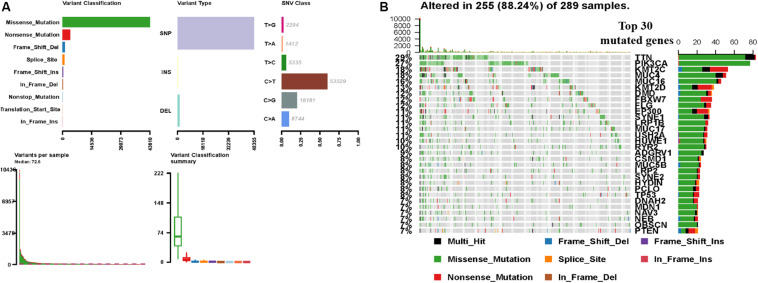
TCGA CCC mutation cohort (*n* = 289). **(A)** Overview of TGCA CCC cohort mutations, including variant classification, variant type, class of SNV, variants in each sample, and variant classification summary. **(B)** Waterfall plot of the top genes commonly mutated in the TCGA CCC cohort, in which the row represents the gene that affects the most frequent mutation in TCGA and the list represents the sample. Different types of genomic changes are represented by different colors. CCC, cervical cell carcinoma. SNV, somatic mutation.

In order to understand the factors related to CCC mutagenesis, we first evaluated and summarized the variation of each sample of TCGA CCC and found that the proportion of Missense Mutation and Single Nucleotide Polymorphism (SNP) was higher. The highest mutation frequency in the sample was 10439 (Figure 1A). The waterfall map showed the integration of somatic mutations in TCGA CCC, in which TTN, PIK3CA, MUC16 and other genes have a higher proportion of mutations, and the highest proportion of Missense Mutation (green) (Figure 1B).

### The Relationship Between TMB and Survival and Clinical Characters

**FIGURE 2:**
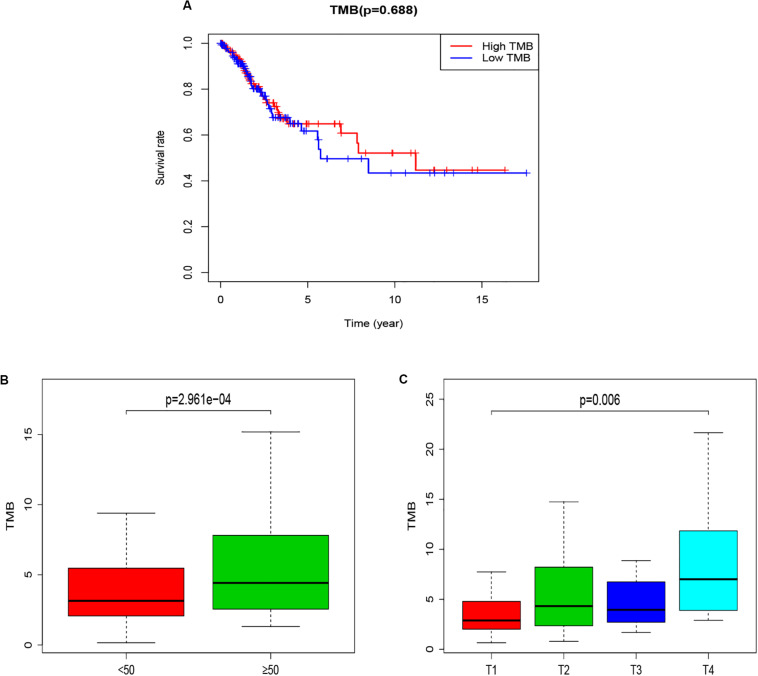
The relationship between TMB and Survival and clinical characters of age and T staging. **(A)** Kaplan-Meier survival curves for high-TMB subtypes (red line) and low-TMB subtypes (blue line). **(B)** Correlation analysis between TMB and age (with 50 years old as the cut-off point). **(C)** Correlation analysis between TMB and T staging. TMB, mutant tumor burden.

The potential correlation of TMB in the prognosis of patients with CCC was explored by Kaplan-Meier method. Patients were divided into low-TMB group and high-TMB group with a TMB median value (3.6579). Although the overall survival time of the high TMB group was not statistically significant compared with the low TMB group (*p* = 0.688), the overall survival time of the high TMB group was higher than that of the low TMB group, especially in 5–10 years (Figure 2A). At the same time, further analysis of the association between TMB and clinical characters, found that the TMB of patients over 50 years old was significantly higher than that of patients under 50 years old (*p* = 2.961e−04, Figure 2B), and the TMB value increased with the increase of T stage (*p* = 0.006, Figure 2C).

### Enrichment Analysis of the DEGs

**FIGURE 3:**
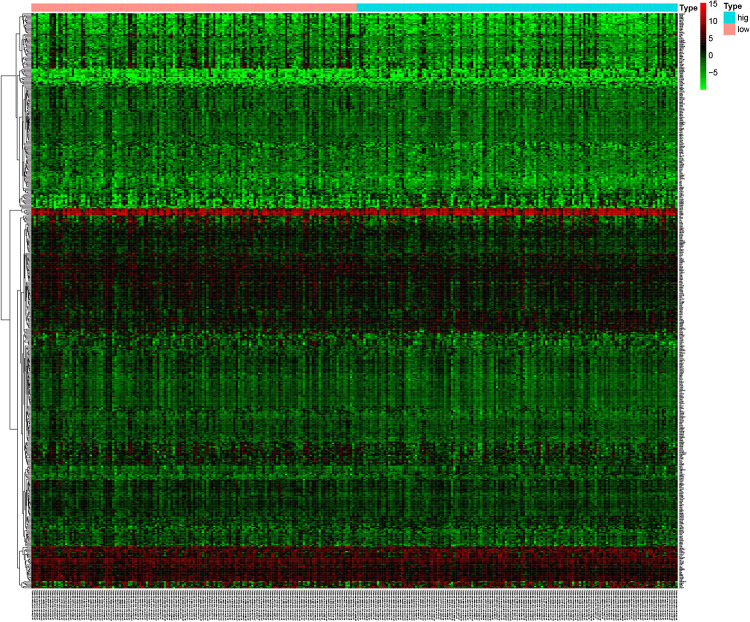
The mutant genes with higher expression in the heatmap are shown in red, lower expression in green, and genes with the same expression level in black. The heatmap of the DEG of the left half (low-TMB subtypes) and the right half (high-TMB subtypes) of TMB. DEG, differentially expressed gene. TMB, mutant tumor burden.

**FIGURE 4:**
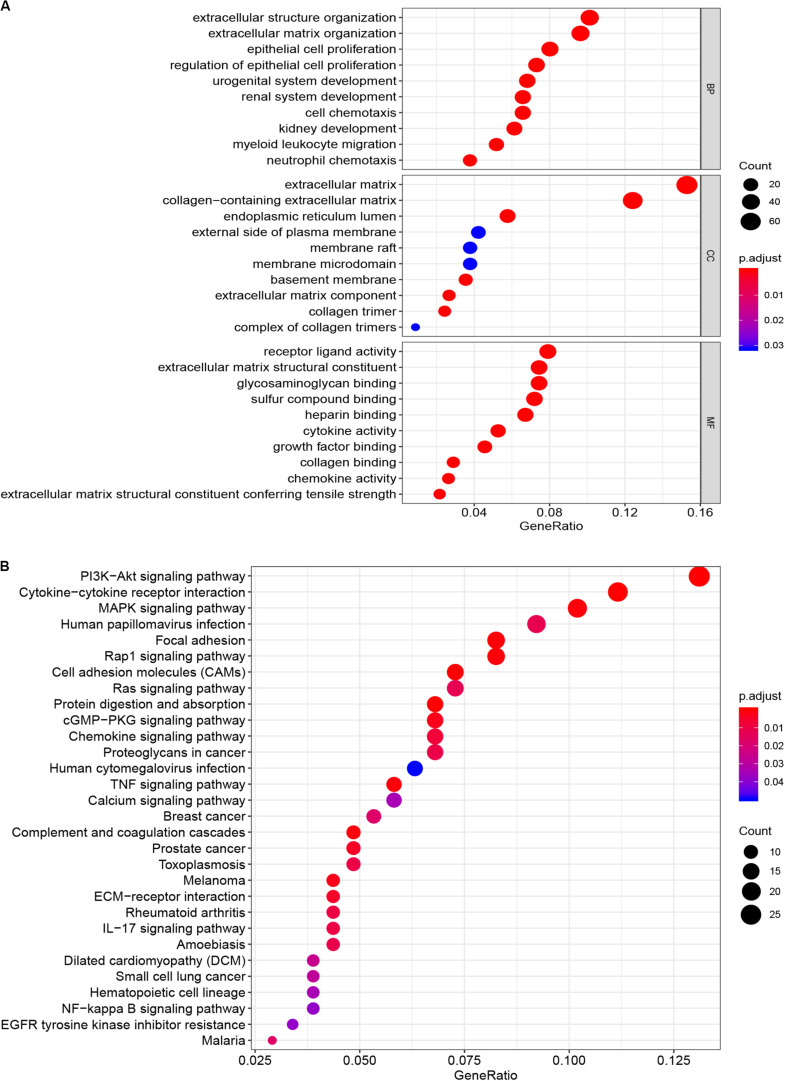
Functional enrichment analysis of DEGs. **(A)** Cellular composition, biological process and molecular function of GO enrichment analysis. **(B)** Enrichment analysis of KEGG pathway. DEG, differentially expressed gene. GO, Gene Ontology; KEGG, Kyoto Encyclopedia of Genes and Genomes.

**FIGURE 5:**
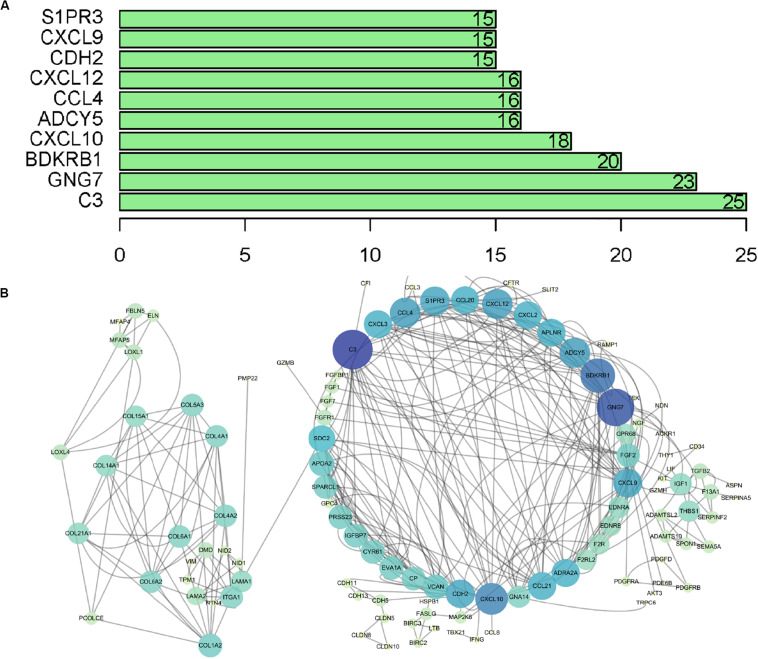
PPI network analysis of DEGs. **(A)** Top 10 core genes according to nodes. **(B)** PPI network visualization, in which the color and size of the map nodes were determined by the degree value with a gradual process. Yellow and small circles represent low degree values, and blue and large circles represent height degree values. PPI, protein–protein interaction.

The microarray dataset of gene expression in TCGA CCC was analyzed by “limma” package, and the DEGs were screened according to *p* < 0.05 and |logFC| > 0.5. The smaller the *p*-value, the greater the possibility of differential gene expression. Finally, we determine 478 DEGs and drew the heatmap of all DEGs with the R-heatmap package (Figure 3). We conducted enrichment analysis to clarify the biological function of DEGs. The GO enrichment analysis showed that most DEGs significantly enrich the extracellular matrix and immune-related cell chemotactic migration (Figure 4A). The first three enriched KEGG pathways are PI3K-Akt, MAPK signaling pathway, cytokine-cytokine receptor interaction and HPV infection (Figure 4B). All of the results are summarized in Tables 1, 2. To further understand the interactions between proteins and better understand their regulatory mechanisms, the DEGs were analyzed using the STRING database and visualized by the Cytoscape software (Figure 5A). According to the degree value, the top ten core genes include C3, GNG7, BDKRB1, CXCL10, ADCY5, CCL4, CXCL12, CDH2, CXCL9, and S1PR3 (Figure 5B).

**TABLE 1:** GO enrichment analysis for DEGs.

Type	Description	BgRatio	*p*-value	*p*.adjust	*q*-value
BP	extracellular matrix organization	348/18493	6.78E–18	2.74E–14	2.12E–14
BP	extracellular structure organization	402/18493	3.83E–17	7.75E–14	5.99E–14
BP	renal system development	286/18493	1.19E–10	1.61E–07	1.24E–07
BP	epithelial cell proliferation	420/18493	2.13E–10	1.98E–07	1.53E–07
BP	cell chemotaxis	295/18493	2.45E–10	1.98E–07	1.53E–07
BP	urogenital system development	322/18493	4.04E–10	2.72E–07	2.11E–07
BP	regulation of epithelial cell proliferation	368/18493	5.20E–10	3.01E–07	2.33E–07
BP	kidney development	271/18493	8.63E–10	4.36E–07	3.37E–07
BP	neutrophil chemotaxis	101/18493	1.23E–09	5.03E–07	3.89E–07
BP	myeloid leukocyte migration	199/18493	1.24E–09	5.03E–07	3.89E–07
CC	extracellular matrix	490/19659	1.49E–34	5.81E–32	5.27E–32
CC	collagen-containing extracellular matrix	332/19659	2.91E–32	5.66E–30	5.13E–30
CC	basement membrane	92/19659	2.88E–10	3.74E–08	3.39E–08
CC	extracellular matrix component	49/19659	7.86E–10	7.64E–08	6.93E–08
CC	endoplasmic reticulum lumen	301/19659	7.90E–09	6.14E–07	5.57E–07
CC	collagen trimer	87/19659	4.79E–06	0.00031	0.000281
CC	external side of plasma membrane	359/19659	0.00068	0.031344	0.028413
CC	membrane raft	304/19659	0.000701	0.031344	0.028413
CC	membrane microdomain	305/19659	0.000727	0.031344	0.028413
CC	complex of collagen trimers	19/19659	0.000806	0.031344	0.028413
MF	extracellular matrix structural constituent	155/17632	3.41E–20	2.03E–17	1.82E–17
MF	heparin binding	163/17632	1.57E–16	4.68E–14	4.18E–14
MF	glycosaminoglycan binding	222/17632	1.53E–15	3.04E–13	2.72E–13
MF	sulfur compound binding	242/17632	1.13E–13	1.68E–11	1.50E–11
MF	growth factor binding	137/17632	5.74E–10	6.85E–08	6.13E–08
MF	cytokine activity	219/17632	1.24E–08	1.23E–06	1.10E–06
MF	chemokine activity	49/17632	1.47E–08	1.26E–06	1.12E–06
MF	receptor ligand activity	478/17632	4.07E–08	3.04E–06	2.72E–06
MF	collagen binding	67/17632	4.84E–08	3.21E–06	2.87E–06
MF	extracellular matrix structural constituent conferring tensile strength	37/17632	1.47E–07	8.77E–06	7.84E–06

*GO, Gene Ontology; DEG, Differentially Expressed Gene; CC, Cellular Composition; BP, Biological Process; MF, Molecular Function.*

**TABLE 2:** KEGG pathway analysis for DEGs.

Description	BgRatio	*p*-value	*p*.adjust	*q*-value
Protein digestion and absorption	90/7843	7.45E–08	1.77E–05	1.38E–05
PI3K-Akt signaling pathway	354/7843	4.96E–07	5.90E–05	4.59E–05
CytokinE–cytokine receptor interaction	294/7843	2.43E–06	0.000192	0.00015
Cell adhesion molecules (CAMs)	146/7843	5.99E–06	0.000357	0.000278
Focal adhesion	199/7843	1.73E–05	0.000821	0.000639
Rap1 signaling pathway	206/7843	2.71E–05	0.00086	0.000669
TNF signaling pathway	110/7843	2.87E–05	0.00086	0.000669
MAPK signaling pathway	295/7843	2.89E–05	0.00086	0.000669
Complement and coagulation cascades	79/7843	3.73E–05	0.000985	0.000767
Melanoma	72/7843	0.000101	0.002412	0.001878
cGMP-PKG signaling pathway	166/7843	0.000112	0.002419	0.001883
Prostate cancer	97/7843	0.000216	0.004279	0.00333
ECM-receptor interaction	82/7843	0.000278	0.005094	0.003965
Chemokine signaling pathway	190/7843	0.000458	0.007778	0.006055
Rheumatoid arthritis	91/7843	0.000607	0.009633	0.007499
IL-17 signaling pathway	93/7843	0.000712	0.01036	0.008065
Toxoplasmosis	113/7843	0.00074	0.01036	0.008065
Proteoglycans in cancer	201/7843	0.000802	0.010609	0.008258
Amoebiasis	96/7843	0.000897	0.011238	0.008748
Human papillomavirus infection	330/7843	0.001045	0.012437	0.009681
Ras signaling pathway	232/7843	0.001129	0.012801	0.009964
Breast cancer	147/7843	0.001639	0.017221	0.013405
Malaria	49/7843	0.001664	0.017221	0.013405
Dilated cardiomyopathy (DCM)	91/7843	0.002596	0.025746	0.020041
Small cell lung cancer	93/7843	0.002976	0.028327	0.02205
Hematopoietic cell lineage	97/7843	0.003863	0.034178	0.026605
Calcium signaling pathway	188/7843	0.003877	0.034178	0.026605
EGFR tyrosine kinase inhibitor resistance	79/7843	0.004559	0.038185	0.029724
NF-kappa B signaling pathway	100/7843	0.004653	0.038185	0.029724
Human cytomegalovirus infection	225/7843	0.006239	0.0495	0.038531

*KEGG, Kyoto Encyclopedia of Genes and Genomes; DEG, Differentially Expressed Gene.*

### Comprehensive Analysis of TMB and Immune Cell

**FIGURE 6:**
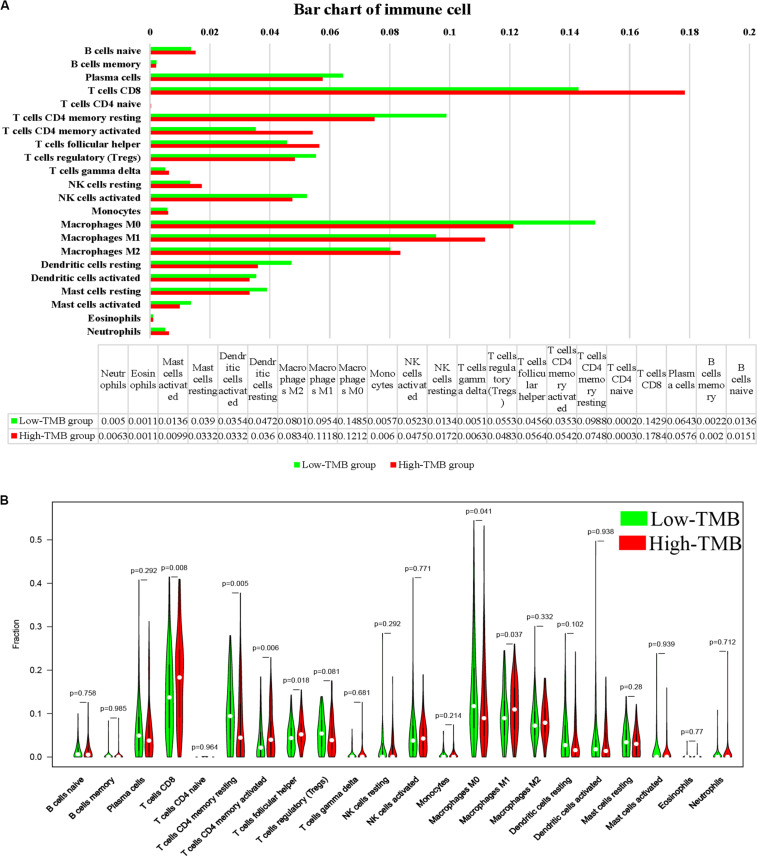
Relationship between the TMB and immune cell. **(A)** The proportion of immune cells in the high- and low TMB subtypes. **(B)** The vioplot contains 22 kinds of immune cells, each of which is shown in red in the high mutation burden group and green in the low mutation burden group. TMB, mutant tumor burden.

The subpopulation analysis of immune cell abundance under the TMB value grouping has not been fully elucidated, so our analysis was based on CCC tumors with at least moderate immune infiltration in terms of cytolytic activity (CIBERSORT, *p* < 0.05). By summarizing and analyzing, the proportion of immune cells in the low-TMB group and the high-TMB group was summarized. The results were shown in Figure 6A, in which the top three immune cells in the low-TMB group were Macrophages M0 (0.1485) and T cells CD8 (0.1429) and T cells CD4 memory resting (0.0988), while the top three in the high-TMB group were T cells CD8 (0.1784), Macrophages M0 (0.1212), and Macrophages M1 (0.1118). By grouping the median TMB value, the 22 immune cell subsets were compared with high and low TMB content to explore the relationship between each immune cell and TMB content. If the *p*-value was less than 0.05, the immune cells are considered to have a significant correlation between high and low TMB group, including T cells CD8, T cells CD4 memory resting, T cells CD4 memory activated, T cells follicular helper, Macrophages M0 and Macrophages M1 (Figure 6B).

### The Prognostic Model of Prognosis-Related Immune Genes

**FIGURE 7:**
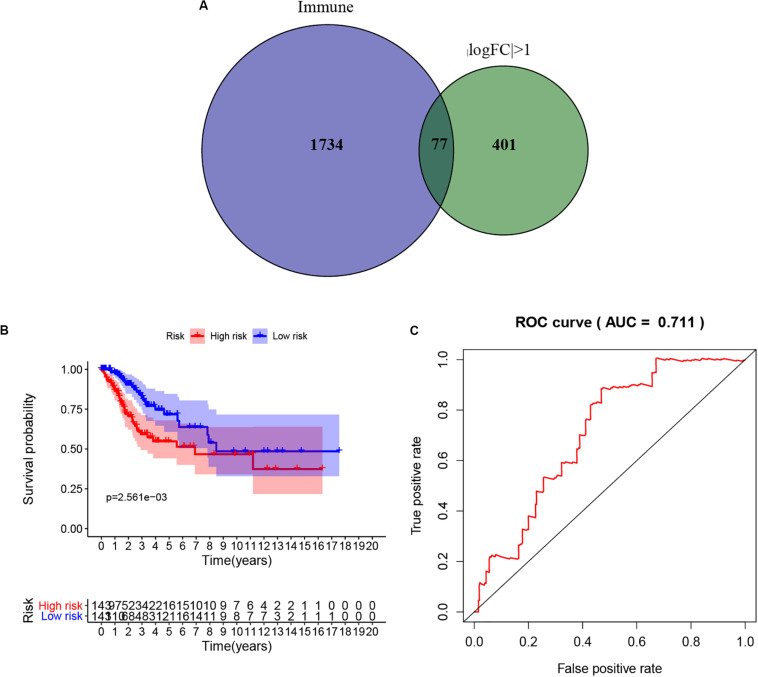
Prognostic model based on the hub TMB-related immune genes. **(A)** Screening of TMB-related immune genes. **(B)** Kaplan-Meier curves of OS for low- and high-risk group (*p* = 0.003). **(C)** ROC curve for judging the accuracy of model predictions (AUC = 0.711). TMB, mutant tumor burden; OS, overall survival; ROC, Receiver operating characteristic.

**FIGURE 8:**
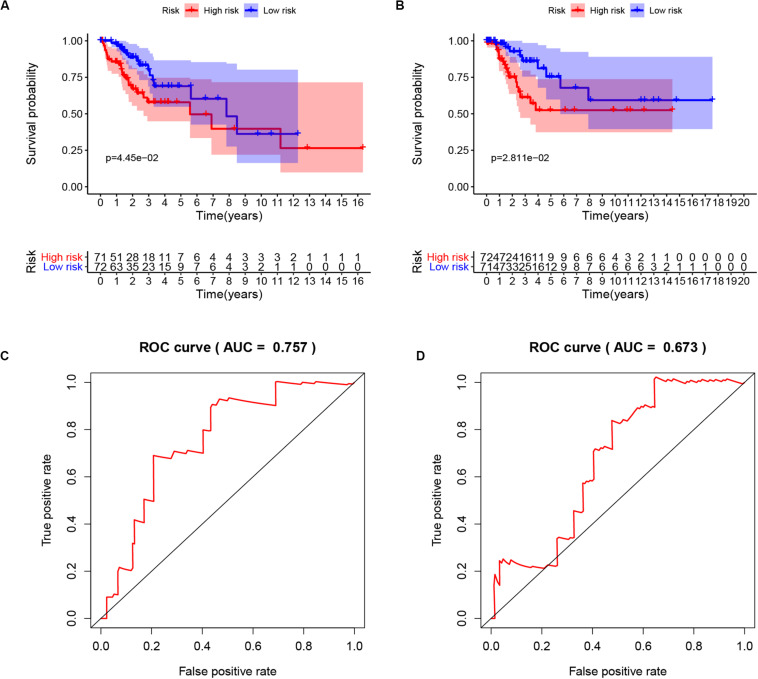
Validation of the TMBPM in the testing set I and testing set II. **(A)** Kaplan-Meier curves of OS of low- and high-risk groups in the testing set I (*p* = 0.0445). **(B)** Kaplan-Meier curves of OS of low- and high-risk groups in the testing set II (*p* = 0.0281). **(C)** ROC curve for judging the accuracy of the TMBPM in the testing set I (AUC = 0.757). **(D)** ROC curve for judging the accuracy of the TMBPM in in the testing set II (AUC = 0.673). TMBPM, TMB Prognostic model; OS, overall survival; ROC, Receiver operating characteristic.

To extract immune genes with greater prognostic efficacy, 77 candidate immune differential genes were subjected to Cox regression analysis (Figure 7A). Based on the multivariate Cox regression model, we obtained TMBPM = (EXP_CLEC3B_ ×−0.35 + EXP_CLEC4A2_ × 0.01). The genes in the TMBPM included CLEC3B and COL4A2 (Table 3). Then, we calculated the risk value for each CCC patient and classified them into high-risk groups (*n* = 143) and a low-risk group (*n* = 143) with a median cut-off value of 1.065. Figure 7B showed that patients in the high-risk group had a lower survival rate than those in the low-risk group (*p* = 0.003), and the AUC of 0.711 indicated that the model has a high prediction accuracy (Figure 7C). This is worth further verification with a large sample. To determine the feasibility and reliability of the TMBPM, we validated the signature in the testing set I (*n* = 143) and testing set II (*n* = 144). In the testing set I and testing set II, the survival of the low-risk group was significantly better than that of the high-risk group (*p* < 0.05) (Figures 8A,B). AUC for the testing set I and the testing set II were 0.757 and 0.673 (Figures 8C,D), respectively, indicating that the signature can better predict the OS of UCEC patients.

**TABLE 3:** Multivariate Cox regression analysis of TMB prognostic model for CCC patients.

ID	Description	Coef	HR (95% CI)	*p*−value
CLEC3B	C-type lectin domain family 3, member B	–0.35438	0.701611 (0.528377–0.93164)	0.01431
COL4A2	collagen, type IV, alpha 2	0.010402	1.010456 (1.003619–1.01734)	0.002675

*TMB, tumor mutation burden; CCC, cervical cell carcinoma; Coef, coefficient; HR, hazard ratio; CI, confidence interval.*

### Immune Infiltrates Analysis of Hub TMB-Related Immune Genes in CCC

**FIGURE 9:**
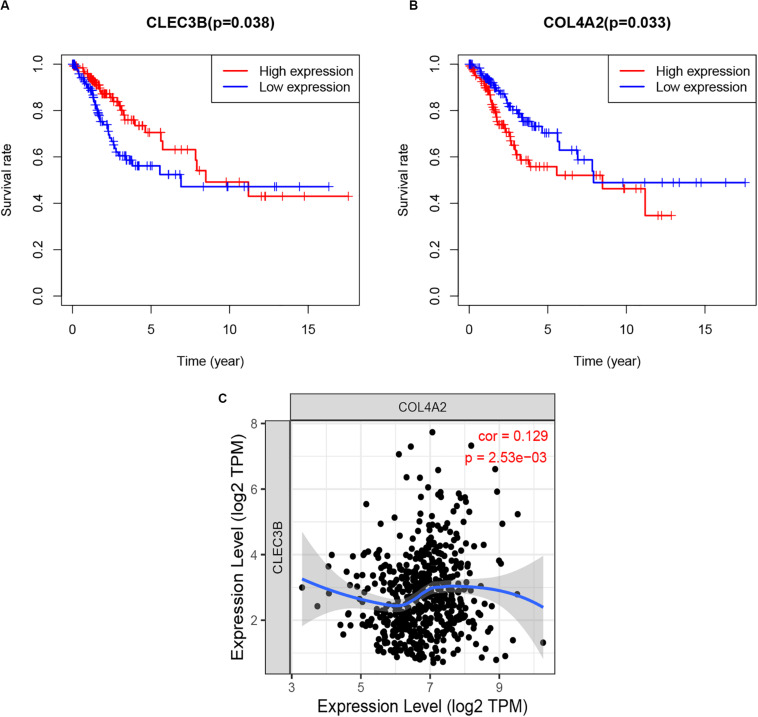
Survival analysis **(A,B)** and correlation analysis **(C)** of two hub TMB-related immune genes with *p*-value. TMB, tumor mutation burden.

As the role of the two hub TMB-related immune genes in immunity is unclear. In the current study, survival analysis using R scripts revealed that higher expression level of CLEC3B was positively associated with poor survival results, whereas COL4A2 expression level was negatively correlated with prognosis (Figures 9A,B), and there was a positive correlation between the expression of two genes (Figure 9C).

**FIGURE 10:**
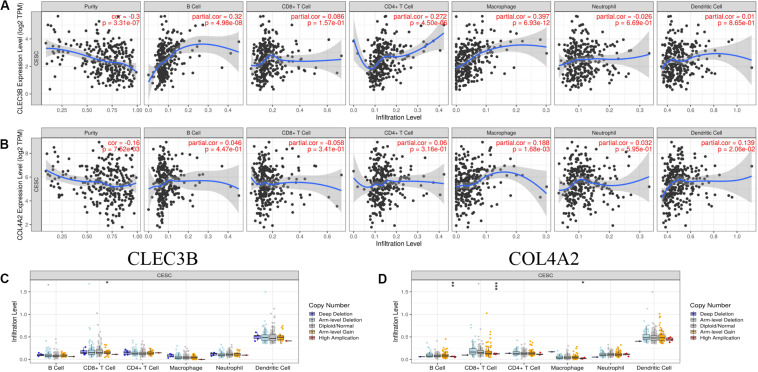
Immune infiltrates analysis of hub TMB-related immune genes in CCC. **(A,B)** The correlation between the immune gene and the immune infiltration level. **(C,D)** Associations of the immune gene mutants with immune cells infiltration. TMB, tumor mutation burden; CCC, Cervical cell carcinoma.

More importantly, we further analyzed the correlations between the abundance of six immune cell types and the expression of these genes, as well as evaluated the mutation types. Interestingly, the expression of CLEC3B was positively correlated with the abundance of B cells (Cor = 0.32, *p* = 4.98e−08), CD4+ T cells (Cor = 0.272, *p* = 4.50e−06), Macrophages (Cor = 0.397, *p* = 6.93e−12) (Figure 10A). As for COL4A2, its expression also was positively correlated with the abundance of Macrophage (Cor = 0.188, *p* = 1.68e−03) and Dendritic cells (Cor = 0.139, *p* = 2.06e−02) was positively correlated (Figure 10B). More importantly, we further evaluated the potential relationship between these two core immune gene mutants and immune infiltration. We observed that mutations in multiple forms of CLEC3B and COL4A typically inhibit immune infiltration, including B cells, CD8+ T cells and Macrophages (Figures 10C,D). These results suggest a possible association between the genes and immune infiltration in CCC.

## Discussion

Cervical cell carcinoma is a common malignant tumor of the female reproductive system, and its incidence has been increasing and younger in recent years, seriously threatening the health of women worldwide. According to the stage of the International Federation of Obstetrics and Gynecology (FIGO), the 5-year overall survival rate of stage II patients was 65∼69%, stage III was 40∼43%, stage IV was 15∼20%, and 29∼38% of the patients had recurrence after treatment, with an overall 5-year survival rate of recurrent patients was only 3.8∼13.0% (Verma et al., 2016).

In recent years, tumor immunotherapy has been widely concerned, especially immune checkpoint inhibitor therapy has entered clinical trials and used in a variety of tumor treatment, but not all patients can benefit from it. In order to find out which patients can benefit, combined with predictive efficacy markers to predict efficacy before immunotherapy, including PD-1, PD-L1, CTLA-4, and TMB (Marin-Acevedo et al., 2018), in which TMB is a promising biomarker in recent years. In general, the higher the TMB, the more mutations there are, the easier it is for immune cells to detect and become the target of tumor immunotherapy, and the more likely it is to be effective for immunotherapy. This part of the study shows that the higher the TMB for all types of cancer, the higher the overall survival rate of patients treated with immune checkpoint inhibitors, which can be used as an additional biomarker of PD-L1. It has been proved that it can be used for a variety of tumors (Samstein et al., 2019), such as small-cell lung cancer (Hellmann et al., 2018), breast cancer and so on (Park et al., 2018). However, its prognostic role and its relevance to immunotherapy in CCC has not been explored, so this study investigated the prognostic role of TMB and the potential relationship between TMB and immune infiltration.

Based on the Kaplan-Meier method, the overall survival trend of patients with high-TMB is higher than that of patients with low-TMB. The reason may be that highly mutated tumors are more likely to carry neoantigens, making them targets for activated immune cells and improving the ability to against cancer cells. Therefore, high tumor mutations were the key mechanism for generating anti-cancer immunity. Similarly, the higher the T stage, the higher the possibility of CCC cell mutation, and the corresponding TMB value tends to increase. Goodman found that patients with high TMB had better progression-free survival (PFS) than those with low TMB for non-small cell lung cancer and melanoma (Goodman et al., 2017). Park similarly found that patients with HER2-positive refractory metastatic breast cancer with high TMB had better overall survival (Park et al., 2018).

At the same time, the GO functional annotation analysis showed that the DEGs was mainly involved in the regulation of extracellular matrix and immune cells. Further analysis of KEGG pathway revealed that these DEGs were associated with CCC mutations, including PI3K-Akt and MAPK signaling pathways. The PI3K/Akt/mTOR signaling pathway was over-activated in liver cancer, colorectal cancer, and esophageal cancer lesions (Wu et al., 2018; Yang et al., 2018). In recent years, studies on CCC cells have confirmed that mTOR inhibitor can inhibit the proliferation and invasion of CCC cells, suggesting that the PI3K/Akt pathway may be involved in the development of CCC (Chang et al., 2017).

We performed subpopulation analysis of immune cells under TMB group, and found that T cells CD8, T cells CD4 memory activated, T cells follicular helper, and Macrophages M1 were all antitumor immune cells and that the overall trend in survival was higher for high TMB (corresponding to high levels of these four immune cells) than for low TMB. Fridman et al. (2011) found that strong local T cells CD8 immune response predicts a better prognosis. The results of Mahmoud et al. (2011) showed that the total number of T cells CD8 in tumors was associated with higher tumor grade and improved patient survival. Similarly, Matsumoto et al. (2016) observed that CD4+ and CD8+ T cells were key factors in tumor immunotherapy and that their high levels of immune infiltration were associated with better clinical outcomes in triple-negative breast cancer. Hammes et al. (2007) found that the number of tumor-associated macrophages in cervical tissue increased with the increase of cervical lesions.

We screened two immune-related DEGs by single- and multifactorial Cox analysis, including CLEC3B and COL4A2. The prognostic model (TMBPM) was developed using these two hub immune genes, and patients with high TMBPM had relatively poor survival outcomes. The AUC of this prognostic model was 0.711, indicating a high predictive accuracy. To our knowledge, this is the first TMBPM to predict CCC survival outcome. However, further large-sample studies are still needed to validate and modify it before clinical application.

The rapid development of tumor immunotherapy in recent years has validated the role of the immune system in the development of cancer. Other important findings of this study were that the expression of CLEC3B and COL4A2 correlated with the level of immune infiltration in CCC. Our results showed that CLEC3B expression was most significantly positive correlated with B cells, CD4+ T cells, and macrophage infiltration. COL4A2 expression was most significantly positive correlated with the presence of macrophage and dendritic cell infiltration. C-type lectin domain family 3 member B (CLEC3B) encodes a tetraspanin that acts by inducing fibrinogen activation, which is associated with tumor invasion and metastasis. CLEC3B has been reported in a variety of tumors, including hepatocellular carcinoma, ovarian cancer, and lung cancer. Dai et al. (2019) reported that CLEC3B downregulation promotes hepatocellular carcinoma metastasis and angiogenesis. Similarly, Sun et al. (2020) found that downregulation of CLEC3B in lung squamous cell carcinoma was associated with poor PFS and OS. There is the most significant positive correlation between CLEC3B and SCC B cells, CD8+ T cells, CD4+ T cells, macrophages and dendritic cells infiltration in lung squamous cell carcinoma. Brown et al. (2015) observed that overexpression of Notch3 correlated with low survival in epithelial ovarian cancer, and the high levels of Notch3 expression in human ovarian tumor specimens correlated with high expression of COL4A2.

There are some shortcomings in this study. There is a lack of basic experiments to verify the predictive efficacy of TMBPM, the correlation between hub immune genes and immune infiltration. Further validation in a larger queue will be required in the future. On the other hand, many immunotherapy methods were introduced in the early stage of CCC, and this study lacked comparison of the prognosis of patients receiving immunotherapy.

## Conclusion

In summary, high TMB may inhibit the development of CCC through anti-tumor immune cells. The prognostic model (TMBPM) based on two immune genes showed that the higher the TMBPM score, the worse the prognosis of the patients. A significant association was obtained between the pivotal immune genes and patient prognosis and immune infiltration, which deserves further validation.

## Data Availability Statement

Publicly available datasets were analyzed in this study. This data can be found here: TCGA.

## Author Contributions

CZ, CL, and HL performed the data analysis work and aided in writing the manuscript. SP designed the study and assisted in writing the manuscript. LZ edited the manuscript. All authors read and approved the final manuscript.

## Conflict of Interest

The authors declare that the research was conducted in the absence of any commercial or financial relationships that could be construed as a potential conflict of interest.

## Footnotes

1 https://portal.gdc.cancer.gov

2 https://www.r-project.org/

3 http://www.bioconductor.org

4 https://www.immport.org/shared/home

5 https://cistrome.shinyapps.io/timer/
